# PDL1 Fusion Protein Protects Against Experimental Cerebral Malaria via Repressing Over-Reactive CD8^+^ T Cell Responses

**DOI:** 10.3389/fimmu.2018.03157

**Published:** 2019-01-14

**Authors:** Jun Wang, Yue Li, Yan Shen, Jiao Liang, Yinghui Li, Yuxiao Huang, Xuewu Liu, Dongbo Jiang, Shuya Yang, Ya Zhao, Kun Yang

**Affiliations:** ^1^Department of Medical Microbiology and Parasitology, Fourth Military Medical University, Xi'an, China; ^2^Department of Immunology, Fourth Military Medical University, Xi'an, China

**Keywords:** PD-1/PDL1, experimental cerebral malaria, macrophage, CD8^+^ T cell, immunotherapy

## Abstract

Cerebral malaria (CM), mainly caused by *Plasmodium falciparum* (*P. f*.), is one of the most lethal complications of severe malaria. As immunopathology mediated by brain-infiltrating CD8^+^ T cells is the major pathogenesis of CM, there is no safe and efficient treatment clinically focused on CD8^+^ T cells. New methods are needed to protect the host from injury. As evidence has shown that programmed death-1 (PD-1) is one of the most efficient immunomodulatory molecules, we constructed two soluble fusion proteins, PDL1-IgG1Fc and PDL2-IgG1Fc, to enhance PD-1/PDL signaling pathways in innate and adaptive immune cells, including macrophages and CD8^+^ T cells. Firstly, we confirmed that PD-1 signal pathway deficiency led to higher levels of CD8^+^ T cell proliferation and shorter survival time in PD-1-deficient (*Pdcd1*^−/−^) mice than WT mice. Secondly, PDL1-IgG1Fc-treated mice exhibited a more prolonged survival time than control groups. Moreover, PDL1-IgG1Fc was observed to ameliorate blood-brain barrier (BBB) disruption by limiting the over-reactive CD8^+^ T cell cytotoxicity during experimental cerebral malaria (ECM). Further studies found thatPDL1-IgG1Fc-treated macrophages showed significant suppression in macrophage M1 polarization and their antigen presentation capability to CD8^+^ T cells. In conclusion, our results demonstrated that the administration of PDL1-IgG1Fc in the early stage before ECM onset has an obvious effect on the maintenance of immune microenvironment homeostasis in the brain and is deemed a promising candidate for protection against CM in the future.

## Introduction

Malaria is still a life-threatening infectious disease worldwide. In 2016, there were an estimated 216 million malaria cases, which led to half a million deaths of children under 5 years old in Africa (1). Cerebral malaria (CM), caused by *Plasmodium falciparum* (*P. f*.), is one of the most dangerous complications of severe malaria (2). Unfortunately, a reliable vaccine is still lacking for the prevention of *P. f*. infection; meanwhile, although the first-line treatments of CM (such as artemisinin-based combination therapies) can produce rapid parasite clearance (3), some patients who receive effective anti-malaria treatment will still die, and even survivors may suffer from long-term neurological sequelae (4).

Regarding pathogenesis, evidence has indicated that parasitized-RBCs (pRBCs) sequestration and vascular immunopathology are the central causes of CM (5), followed by blood-brain barrier (BBB) disruption, neurological damage, and fatal brain edema accompanied by cerebral petechial hemorrhages (6). Therefore, it is crucial to control immunopathology to protect against CM. However, although regular adjunctive therapy in a clinic (such as corticosteroid therapy) can alleviate an over-reactive host immune response, the patients unavoidably suffer from severe side-effects (7). Therefore, a novel adjunctive immunotherapy against pathology during CM is imperative.

Abundant evidence has demonstrated that parasite-specific brain-infiltrating CD8^+^ T cells are the prime culprit for vascular immunopathology during ECM (8–12). To limit pathogenic CD8^+^ T cell immunity, we identified the PD-1/PDL1 pathway as a promising target. PD-1 is broadly expressed on activated T cells, regulatory T cells, and other hematopoietic cells and contains an immunoreceptor tyrosine-based inhibitory motif (ITIM) in its cytoplasmic domain (13). PDL1 and PDL2 are B7-family molecules that bind to PD-1 and down-regulate T cell activation (14). As PD-1 blockade therapy has achieved major breakthroughs in cancer treatment, it is a strong inspiration for us that proper enhancement of the PD-1/PDL negative signaling pathway with exogenous PDLs may attenuate CD8^+^ T cell activation and protect against ECM.

Recent studies have mostly focused on chronic malarial infections by repressing the PD-1/PDL pathway. Instead, for the first time, we enhanced the PD-1/PDL pathway using PDL fusion proteins in acute lethal malaria infection. PDL fusion proteins contain a mouse PDL1 or PDL2 extracellular fragment and an IgG1Fc fragment. We found that PDL1-IgG1Fc notably improved the survival rate of experimental cerebral malaria (ECM) mice via direct inhibition of CD8^+^ T cell functions. Additionally, macrophage M1 polarization and its antigen presentation capability were also suppressed in PDL1-IgG1Fc-treated ECM mice. The results demonstrated that PDL1-IgG1Fc may be a promising candidate for CM adjunctive immunotherapy.

## Materials and Methods

### Ethics Statement

All animal experiments were approved by the Institutional Review Board of the Fourth Military Medical University (No: 20160107) and were in complete compliance with the principles of ARRIVE guidelines for reporting animal research (15). All efforts were made to minimize suffering of animals employed in this study.

### Mice and Parasites

C57BL/6 male mice and *Pdcd1*^−/−^ C57BL/6 male mice (6–8 weeks old) were used for infection experiments. *Pdcd1*^−/−^ mice were kindly provided by Dr. T. Honjo, and all mice were bred and housed under specific pathogen-free conditions in the animal center of FMMU. *Plasmodium berghei* ANKA strain (PbA) was maintained and used as previously reported in our laboratory. All mice were infected intraperitoneally (i. p.) with 1 × 10^7^ pRBCs. Parasitemia was determined by Giemsa-stained thin blood smears. Mice were monitored daily for body weight and symptoms of ECM. For experiments with multiple groups, all mice were first infected, and then randomly assigned into treatment groups.

### Antibodies and Reagents

Antibodies against mouse CD3 (SP7, ab16669), CD28 (PV-1, ab25234), and CD31 (ab28364) were purchased from Abcam. APC-labeled antibodies against mouse PD-1 (29F.1A12, 135209) and CD31 (390, 102409) were purchased from Biolegend. IL-1β (DKW12-2012-096), TNF-α (DKW12-2720-096), IFN-γ (DKW12-2000-096), IL-10 (DKW12-2100-096), and TGF-β (DKW12-2710-096) were measured using murine ELISA kits from DAKEWEI. CFSE Cell Division Tracker Kit was purchased from Biolegend. Recombinant mouse IFN-γ (50709-MNAH) and M-CSF (51112-MNAH) were purchased from SinoBiological. Cell Counting Kit (C008-3) was purchased from 7 Sea Pharmatech. LDH Cytotoxicity Assay Kit was purchased from Beyotime Biotechnology. LPS (L2630) was purchased from Sigma-Aldrich. FITC-BSA (bs-0292P-FITC) was purchased from Biosynthesis Biotechnology. A MILLIPLEX MAP KIT (MCYTOMAG-70K) was purchased from Merck Millipore. All these antibodies and reagents were used in the schedules and doses indicated.

### BMECs Primary Culture

The technique for isolating mouse BMECs was adapted from published protocols (16). Mice were euthanized and perfused with saline. And brains were finely minced with 1 ml of medium and homogenized by passing through a 23-gauge needle. The homogenate was mixed with an equal volume of 30% dextran (MW 70,000, BBI) in PBS and centrifuged at 10,000 g for 15 min at 4°C. The pellet was resuspended in PBS and passed through a 40 μm cell strainer that retained the microvessels. After washing, the cell strainer was back-flushed with 2 ml PBS over a 6-well plate to collect the microvessels, which were rocked at room temperature with 2% FBS, 1 mg/ml collagenase II (02100502, MP Biomedicals) and 20 μg/ml DNase I (10104159001, Sigma-Aldrich) for 90 min. Vessel fragments were collected and resuspended in EC medium (0.1 mg/ml EC growth supplement from ScienCell, catalog #1001) with 4 μg/ml puromycin and seeded into a collagen-coated 6-well plate. The medium was replaced (without puromycin) 3 days later and every 3–4 days thereafter. The purity of BMECs was identified with CD31 by flow cytometry. For cytokine activation of BMECs, 20 ng/ml IFN-γ was added to the cell medium 24 h prior to subsequent analysis.

### Purification of Brain-Sequestered Leukocytes (BSLs) and CD8^+^ T Cells

Mice infected with pRBCs 7 dpi were euthanized and perfused with saline to remove non-adhered RBCs and leukocytes from the brain. Brains were removed, cut into small pieces and crushed in RPMI medium; the brain homogenates were centrifuged at 250 g for 10 min at 4°C, the pellets were dissolved in RPMI medium containing 1 mg/ml collagenase II and 10 μg/ml DNase I for 30 min at 37°C. Cell debris was removed by pushing the mixture through a 40 μm cell strainer. The tissue extract was then centrifuged at 400 g for 5 min. The pelleted cells were further purified on a 30% Percoll gradient (17-0891-02, GE Healthcare). The upper Percoll layers were carefully removed, and the cell pellet resuspended in PBS. The pellet was resuspended in RBC lysis buffer and incubated on ice for 5 min to lyse adherent pRBCs. BSLs were resuspended in PBS and counted. CD8^+^ T cells were negatively isolated from BSLs according to the manufacturer's instructions (558471, BD).

### EC Leakage Assay

To detect the cytotoxicity of activated CD8^+^ T cells to brain endothelial cells, we constructed a BBB model *in vitro* with the bEnd.3 endothelial cell line. The cells (2 × 10^4^) were seeded onto the upper chamber of a 24-well Transwell system (0.4 μm, CLS3450-24EA, Corning). Transwell was checked for the formation of an intact monolayer on the insert by adding FITC-BSA (50 μg/ml) to the upper chamber and measuring the amount of FITC-BSA that passed into the lower chamber. The Transwells were used only when the intensity of fluorescence in the lower chamber was negligible, and bEnd.3 cells were stimulated with IFN-γ (20 ng/ml) and parasites (3 × 10^6^ pRBCs) 24 h. bEnd.3 were washed, and 1 × 10^6^ activated CD8^+^ T cells from PbA-infected mice were added. The extent of BBB damage by CD8^+^ T cells is reflected by the diffusion rate of the FITC-BSA.

### *Ex vivo* Killing Assays of CD8^+^ T Cells Against BMECs

BMECs were isolated from uninfected C57BL/6 mice as described above for an *ex vivo* cell-killing assay. BMECs were activated with IFN-γ (20 ng/ml) and co-incubated with pRBCs for 24 h. Then, the BMECs were incubated at various effector:target (E:T) ratios with activated/naïve CD8^+^ T cells. The cell culture supernatants were collected, and LDH release cytotoxicity assays were carried out to detect the cytotoxicity of CD8^+^ T cells for an LDH content assay. Moreover, granzyme B in the supernatants was determined using ELISA kits.

### Macrophage-CD8^+^ T Cell Co-incubation Model

Bone marrow-derived macrophages were planted into 6-well cell culture clusters and stimulated with a sub-optimal concentration of IFN-γ (0.5 ng/ml), (Figure S4) 1 × 10^7^ pRBCs were subsequently added. Next, these wells were divided into three groups, adding PDL1-IgG1Fc and IgG1Fc as well as cell culture medium as controls. After 24 h incubation, the above mentioned stimulating factors, such as IFN-γ, pRBCs, and soluble fusion proteins were washed away via replacing the culture medium. Then, splenic CD8^+^ T cells were added to the above wells. After 24 hincubation, CD8^+^ T cells were collected, and the expression of perforin and granzyme B were detected through qPCR.

### CD8^+^ T Cell Proliferation Assay

Splenic CD8^+^ T cells were negatively isolated from PDL1/PDL2-IgG1Fc-treated or IgG1Fc-treated mice by magnetic sorting (558471, BD). CD8^+^ T cells were resuspended at 1 × 10^7^ cells/ml in the 5 μM CFSE working solution; the cells were incubated for 20 min at 37°C and protected from light, and then the staining was stopped by adding 5 times the original staining volume of cell culture medium containing 10% FBS. CFSE-labeled cells were harvested, sub-optimal concentrations of anti-CD3 and anti-CD28 mAbs (0.03 μg/ml) were added to the culture system for 4 days, proliferation was assessed by CFSE dilution with flow cytometry and analyzed by Flowjo software.

### BBB Integrity Assay

*In vivo* BBB permeability was checked by an EB assay. Mice were injected by intravenous (i. v.) with 100 μl of 1% EB in PBS. After 8 h, the mice were lethally anesthetized and perfused with saline to remove intravascular EB. Each brain was removed, weighed, and incubated in 0.5 ml formamide overnight at 37°C. The amount of extracted EB was determined by a photometric analysis of the EB-formamide solution at 620 nm and by comparison to an EB standard curve. EB showed red fluorescence under a fluorescence microscope, which could reflect BBB damage *in situ*.

### Brain Water Content Assay

Brain water content was measured as previously published. Weights of brains removed at the indicated time points were compared with the dry weight after overnight incubation at 80°C.

### Histology

To prepare paraffin sections of the brain, mice were perfused with saline. Brains were fixed in 4% paraformaldehyde (PFA) overnight. Dehydration was carried out using sequential alcohol washes with 30, 50, 70, 90, and 100% ethanol. Xylene was used to perforate the brain tissue. The brains were molded using paraffin wax, and 5 μm tissue sections were prepared, collected on poly-L-lysine-coated slides, and stained with H&E. Slides were checked under a light microscope to capture images.

### Immunofluorescence Staining

10 μm-thick frozen sections were fixed in 4% PFA and blocked with 10% goat serum. Sections were incubated with unlabeled specific primary antibodies for 90 min at room temperature (isotype-matched antibodies used as controls) and with fluorescence-labeled secondary antibodies for 30 min at room temperature. Immunofluorescence was examined by fluorescence microscope (Olympus BX51).

### Fusion Proteins

We prepared the ECDs of PDL1 and PDL2 and constructed fusion proteins with the ECDs linked to the hinge-CH2-CH3 domains of murine IgG1. Primers that were designed and synthesized based on the published sequence:

**Table:** 

**PDL1**	**5′-CAGAGATCTATGAGGATATTTGCTGGCATT-3′**
	**5′CTCGAATTCGTGAGTCCTGTTCTGTGGAGG-3′**
**PDL2**	**5′-CAGAGATCTATGCTGCTCCTGCTGCCGATA-3′**
	**5′-ATCGAATTCCCACGTTCTGGGGACTTTGGG-3′**
**IgG1Fc**	**5′GACGAATTCGTGCCCAGGGATAGTGGTAGTAAGCCTAGCATAAGTACAGTCCCAGAAGTATCATCT-3′**
	**5′-ATTCCGCGGTCATTTACCAGGAGAGTGGGA-3′**

cDNAs encoding the mouse PDL1/PDL2 extracellular domain and IgG1Fc were generated by RT-PCR from mouse T cell mRNA. Cysteine in the hinge region was replaced by serine. After digestion with EcoR I and Bgl II and gel purification, the PCR products were fused to the Fc domain of mouse IgG1Fc. The recombinant plasmids were transfected into the FreeStyle 293 expression system (ThermoFisher, K900010) and produced as described. Then, the proteins were purified directly from cell culture supernatants harvested by protein A-Sepharose. The protein concentrations were measured by absorbance at 280 nm, and we used SDS-PAGE and western Blot to confirm purity.

PD-1/PDL signaling can inhibit the TCR-mediated proliferation of T cells. Therefore, the effect of the PDL1/PDL2 fusion proteins was evaluated by their ability to inhibit CD8^+^ T cell proliferation stimulated by anti-CD3 and anti-CD28 mAbs.

### Statistical Analyses

Statistical analyses were performed using PRISM GraphPad™ software (La Jolla, CA, USA) and included Student's *t*-test; *p*-values <0.05 were considered to be significant.

## Results

### The PD-1 Signal Pathway Participates in ECM Development

**Figure 1:**
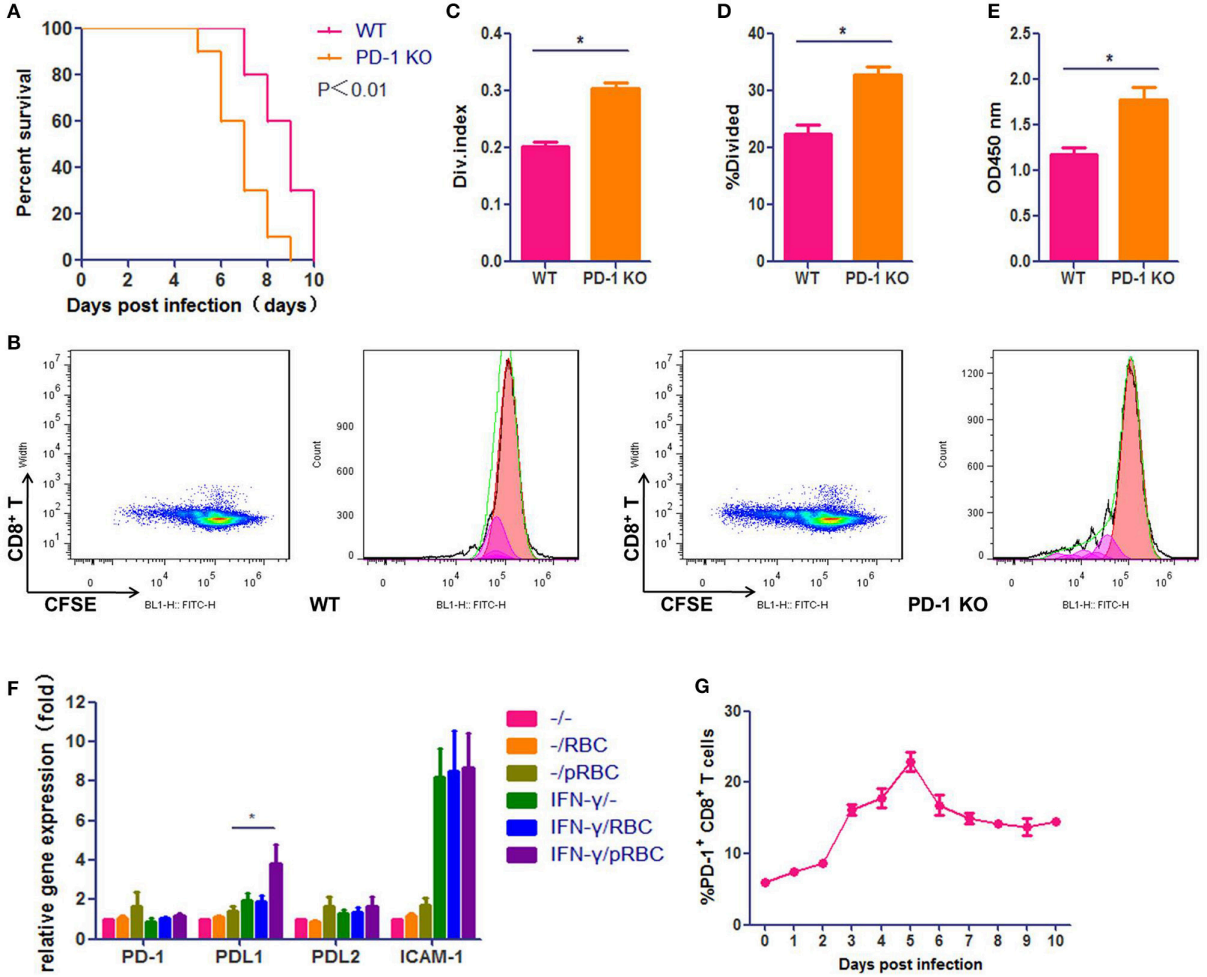
The PD-1 signal pathway participates in ECM development. C57BL/6 male mice and *Pdcd1*^−/−^ C57BL/6 male mice (6–8 weeks old) were infected i. p. with 1 × 10^7^ pRBCs. Survival was monitored daily **(A)**. *Pdcd1*^−/−^ mice showed exacerbated symptoms of ECM, and their survival time was shorter than that of WT mice (log-rank test, *n* = 10, *P* = 0.0046). Splenic CD8^+^ T cells were isolated from infected *Pdcd1*^−/−^ and WT mice 5 dpi, co-incubation with sub-optimal concentrations of anti-CD3 and anti-CD28 mAbs (0.03 μg/ml) after CFSE staining. After 4 days of culture. Results of flow cytometry **(B)**, divided index **(C)** and percentage of divided cell **(C)** analyzed by Flowjo software showed higher proliferation ability of CD8^+^ T cells in *Pdcd1*^−/−^ mice than in WT mice. Proliferation assay used by CCK-8 showed the same results **(E)**. **(F)** qPCR showed that the expression levels of PDL1 and ICAM-1 were highly increased in primary BMECs stimulated by IFN-γ and pRBCs. **(G)** The ratio of splenic PD-1^+^ CD8^+^ T cells to total CD8^+^ T cells was monitored daily in ECM mice. Results are from three independent experiments. Data are from three independent experiments.^*^ indicates that differences are significant (unpaired *t*-test, *n* = 10, 0.01 < *P* < 0.05).

We used the well-characterized PbA infection of C57BL/6 mice as the ECM model, which was confirmed to be credible in our laboratory (Figure S1). To verify the importance of the PD-1 pathway in ECM development, we used PbA-infected *Pdcd1*^−/−^ C57BL/6 mice as the ECM model. We found that *Pdcd1*^−/−^ mice showed earlier neurological signs of ECM than WT mice, and the median survival time of *Pdcd1*^−/−^ mice was 7 days, which was 2 days shorter than that of WT mice (Figure 1A). PD-1 pathway deficiency led to higher levels of T cell proliferation detected by CFSE assay (Figures 1B–D) and Cell Counting Kit (CCK-8) assay (Figure 1E). Then, we evaluated the expression of PD-1, PDL1, and PDL2 by brain microvascular endothelial cells (BMECs). BMECs were either untreated or activated with IFN-γ and pRBCs to mimic the pro-inflammatory microenvironment typically observed in the brain of ECM mice. PD-1, PDL1, and PDL2 mRNA expression levels in BMECs were examined by qPCR (Figure 1F). PDL1 was induced 4-fold at 24 h, while PD-1 and PDL2 showed little up-regulation. In addition, the expression of ICAM-1 was highly up-regulated in the activated BMECs. Furthermore, we detected the ratio of splenic PD-1^+^ CD8^+^ T cells to total CD8^+^ T cells in ECM mice from 0 day post-infection (dpi) to 10 dpi (Figure 1G) and found that the ratio of PD-1^+^ CD8^+^ T cells rose after infection (6.0%) and peaked (22.8%) at 5 dpi. All these results confirmed that the PD-1pathway participates in ECM development and that PDL1 may play a more important role than PDL2 does.

### Activated CD8^+^ T Cells Kill BMECs During ECM

**Figure 2:**
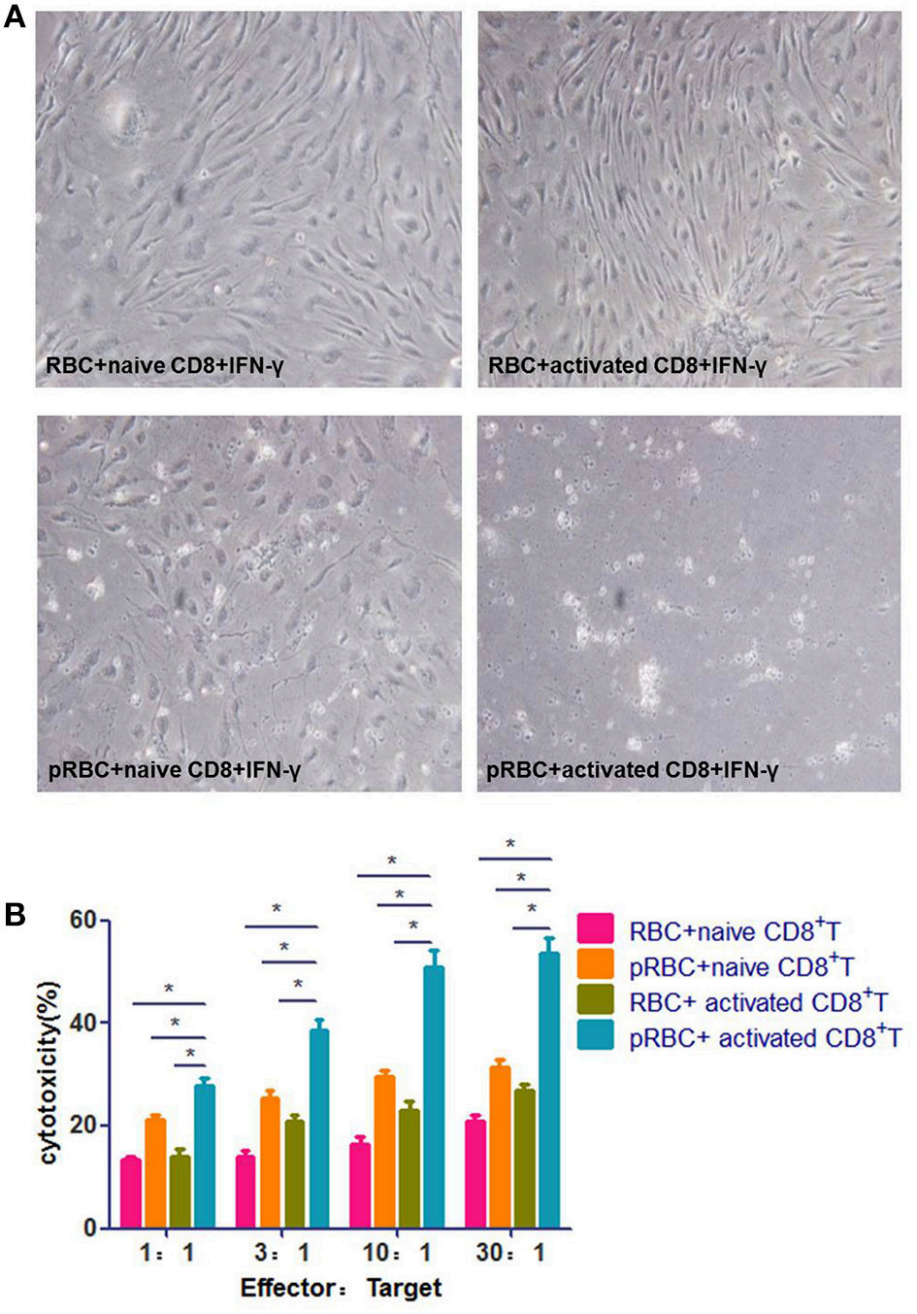
Activated CD8^+^ T cells kill BMECs during ECM. **(A)** BMECs were stimulated by IFN-γ and co-incubation with/without pRBCs for 24 h and then, CD8^+^ T cells were added, which were from either a naïve mouse or a mouse infected with PbA 7 dpi. BMECs were photographed after 24 h. **(B)** BMECs were incubated at various E:T ratios with activated/naïve CD8^+^ T cells. Cell culture supernatants were collected for an LDH-release cytotoxicity assay. Results are from three independent experiments.^*^ indicates that differences are significant (unpaired *t*-test, 0.01 < *P* < 0.05).

The prevailing ECM model is characterized by a severe disruption of BMECs, which is caused by PbA-specific CD8^+^ T cells. To test this model, we added activated splenic CD8^+^ T cells, which were isolated from a PbA-infected mouse immediately prior to ECM (7 dpi), to BMECs that stimulated with IFN-γ and pRBCs. The BMECs were almost entirely dead after 24 h co-incubation. When pRBCs was replaced with RBCs, the activated CD8^+^ T cells did not kill BMECs (Figure 2A). When naïve CD8^+^ T cells from an uninfected mouse were added to PbA-stimulated BMECs, BMECs showed some dysfunction caused by interaction with parasite antigens. Hence, this *in vitro* experiment confirmed that BMECs could be recognized and killed by the activated CD8^+^ T cells. Additionally, BMECs were incubated at various E:T ratios with naïve or activated CD8^+^ T cells, and co-culture supernatants were collected; an LDH-release cytotoxicity assay also showed profound cell damage of BMECs. These data indicated that, during ECM, the activated CD8^+^ T cell is the main contributor that kills BMECs (Figure 2B).

### PDL1-IgG1Fc Treatment Protects Mice From ECM

Based on the results mentioned above, we hypothesize that enhancement of the PD-1 pathway may down-regulate the inflammatory response and the cytotoxic effect of CD8^+^ T cells on BMECs. We constructed two soluble fusion proteins, PDL1-IgG1Fc and PDL2-IgG1Fc, which were confirmed by western blot (Figure S2A). The biologic activities of the fusion proteins were demonstrated by their inhibitory effect on the CD8^+^ T cells proliferation stimulated by sub-optimal concentrations of anti-CD3 and anti-CD28 mAbs. PDL1-IgG1Fc inhibited the proliferation of CD8^+^ T cells in a dose-dependent fashion relative to the IgG1Fc control; in contrast, PDL2-IgG1Fc had no effect on the proliferation of CD8^+^ T cells under the same conditions. There was no obvious synergistic effect between the two recombinant proteins (Figures S2B,C). These results showed that the PDL1-IgG1Fc fusion protein could attenuate the T cell proliferation stimulated by anti-CD3 and anti-CD28 mAbs.

**Figure 3:**
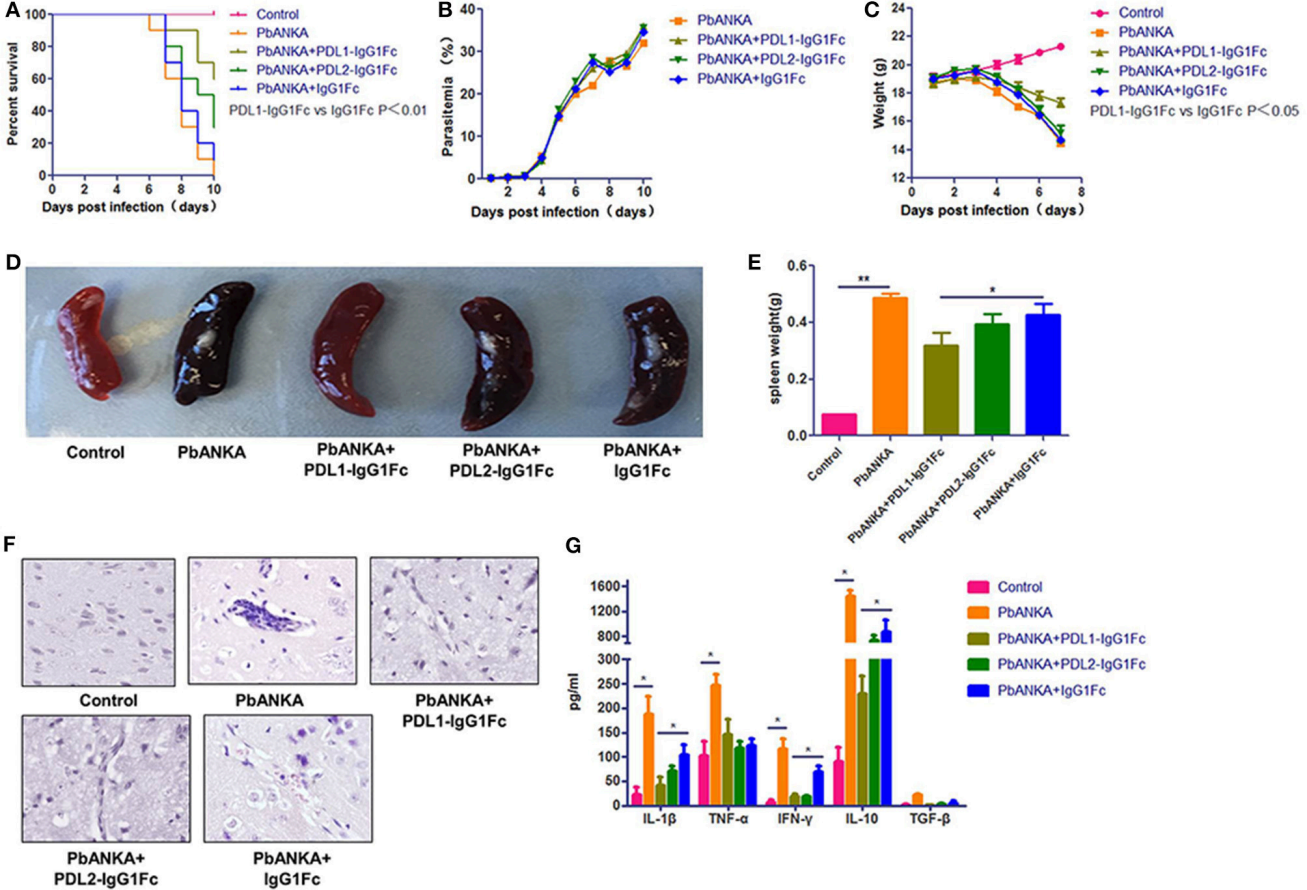
PDL1-IgG1Fctreatment protects mice from ECM. C57BL/6 mice were infected with PbA and injected i. v. with PDL1-IgG1Fc/PDL2-IgG1Fc (0.1 mg/per mouse) or IgG1Fc (0.05 mg/per mouse) on day 0. Survival curves **(A)** (log-rank test, *n* = 10, *P* = 0.001), Blood parasitemia **(B)**. Body weight change **(C)**, Splenomegaly **(D,E)**, H&E-stained brain sections **(F)**, Serum IL-1β, TNF-α, IFN-γ, IL-10, and TGF-β concentrations **(G)** at 7 dpi demonstrated that PDL1-IgG1Fc treatment protected mice from ECM. Data are from at least three independent experiments. ^*^ and ^**^ indicate that differences are significant (unpaired *t*-test, *n* = 10, 0.01 < *P* < 0.05 and 0.001 < *P* < 0.01, respectively).

Next, to determine whether PDL1/PDL2-IgG1Fc could modulate malaria pathogenesis, C57BL/6 mice were infected with PbA and treated with PDL1/PDL2-IgG1Fc and IgG1Fc at day 0. Body weight, parasitemia and survival were monitored daily. The PDL1-IgG1Fc treatment was most effective in delaying the onset of ECM, and compared with PDL2-IgG1Fc-treated and IgG1Fc-treated mice, PDL1-IgG1Fc-treated mice displayed prolonged survival times (Figure 3A), reduced body weight loss (Figure 3C) and splenomegaly (Figures 3D–E), although there was no obvious difference in peripheral parasitemia between PDL1-IgG1Fc-treated mice and PbANKA group (Figure 3B). Moreover, brain sections were analyzed histopathologically following H&E; consistent with previous results, mice treated with PDL1-IgG1Fc exhibited markedly less cerebral edema, fewer pRBCs sequestrations and less lymphocyte infiltration than PbANKA group (Figure 3F). These findings confirm that PDL1-IgG1Fc can protect mice from ECM.

Furthermore, ECM is associated with excessive systemic inflammation, which involves pro-inflammatory cytokine production that leads to BMECs activation and vascular permeability. In the serum of PbANKA group pro-inflammatory cytokines, including IFN-γ, TNF-α, and IL-1β, rose at day 7, as did anti-inflammatory cytokine IL-10. The levels of serum pro-inflammatory cytokines, including IL-1β, and IFN-γ, in PDL1-IgG1Fc-treated mice were much lower than those in IgG1Fc-treated mice; besides, the levels of anti-inflammatory cytokines, such as IL-10 were also reduced in PDL1-IgG1Fc-treated mice compared with those in PDL2-IgG1Fc-treated and IgG1Fc-treated mice (Figure 3G). Nevertheless, the levels of serum TGF-β were barely detectable in all groups of mice. In summary, PDL1-IgG1Fc treatment protected mice from ECM via relieving the systemic inflammation.

### PDL1-IgG1Fc Reduces BBB Damage Caused by ECM

**Figure 4:**
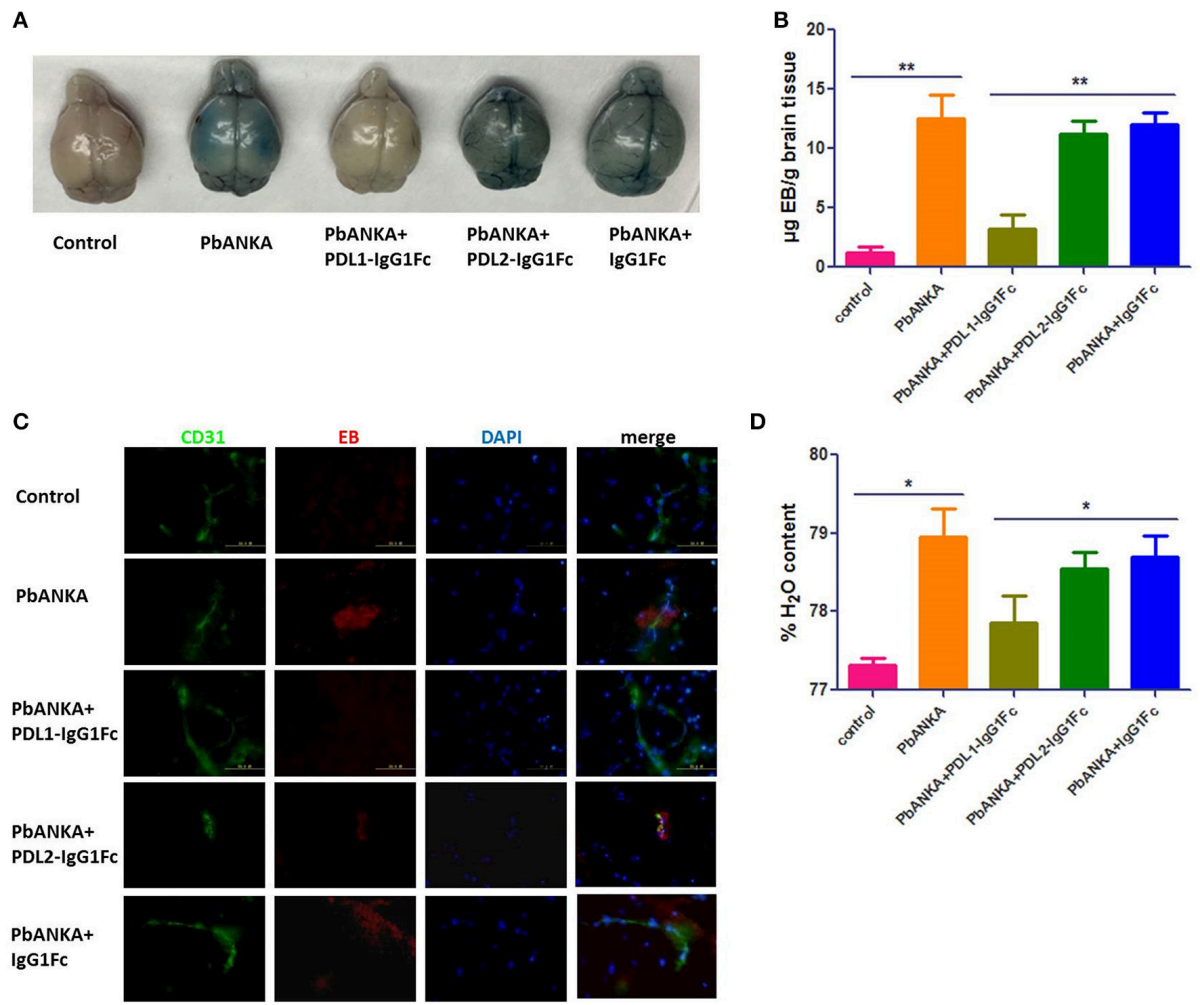
PDL1-IgG1Fc reduces BBB damage caused by ECM. **(A)** Representative brains from PDL1/PDL2-IgG1Fc-treated mice injected i. v. with 100 μl of 1% EB at 7 dpi. **(B)** Spectrophotometric quantification of EB. **(C)** Fluorescence of EB showed less BBB damage in PDL1-IgG1Fc-treated mice (Green: CD31, Red: fluorescence of EB, Blue: nucleus). **(D)** Quantification of brain water content showed that PDL1-IgG1Fc protected mice from vascular leakage. Results are from three independent experiments. ^*^ and ^**^ indicate that differences are significant (unpaired *t*-test, *n* = 10, 0.01 < *P* < 0.05 and 0.001 < *P* < 0.01, respectively).

As BBB damage is the most important pathogenetic mechanism of ECM, we used an Evans blue (EB) permeability assay to evaluate BBB integrity. When the BBB is intact, EB cannot enter the brain parenchyma, after BBB breakdown, EB can enter the brain and shown the localization of BBB injury. The integrity of the BBB was compromised in PbANKA group, as demonstrated by EB leakage into the brain parenchyma at 7 dpi. Mice of PbANKA group were injected intravenously with EB 8 h before being killed, and the EB penetration was notably alleviated in PDL1-IgG1Fc-treated mice compared with that in PDL2-IgG1Fc-treated and IgG1Fc-treated mice (Figure 4A). The spectrophotometric quantification of EB revealed nearly 4-fold less EB in PDL1-IgG1Fc-treated mice than in other mice (Figure 4B). The fluorescence of EB in the cerebral cortex showed less BBB damage in PDL1-IgG1Fc-treated mice, with fewer BBB damage (Figure 4C). PDL1-IgG1Fc-treated mice showed less cerebral edema than did other mice (Figure 4D). All these results confirmed that PDL1-IgG1Fc plays a protective role against BBB damage during ECM.

### PDL1-IgG1Fc Ameliorates BBB Disruption by Limiting the Targeted Killing of BMECs by Over-Reactive CD8^+^ T Cells

**Figure 5:**
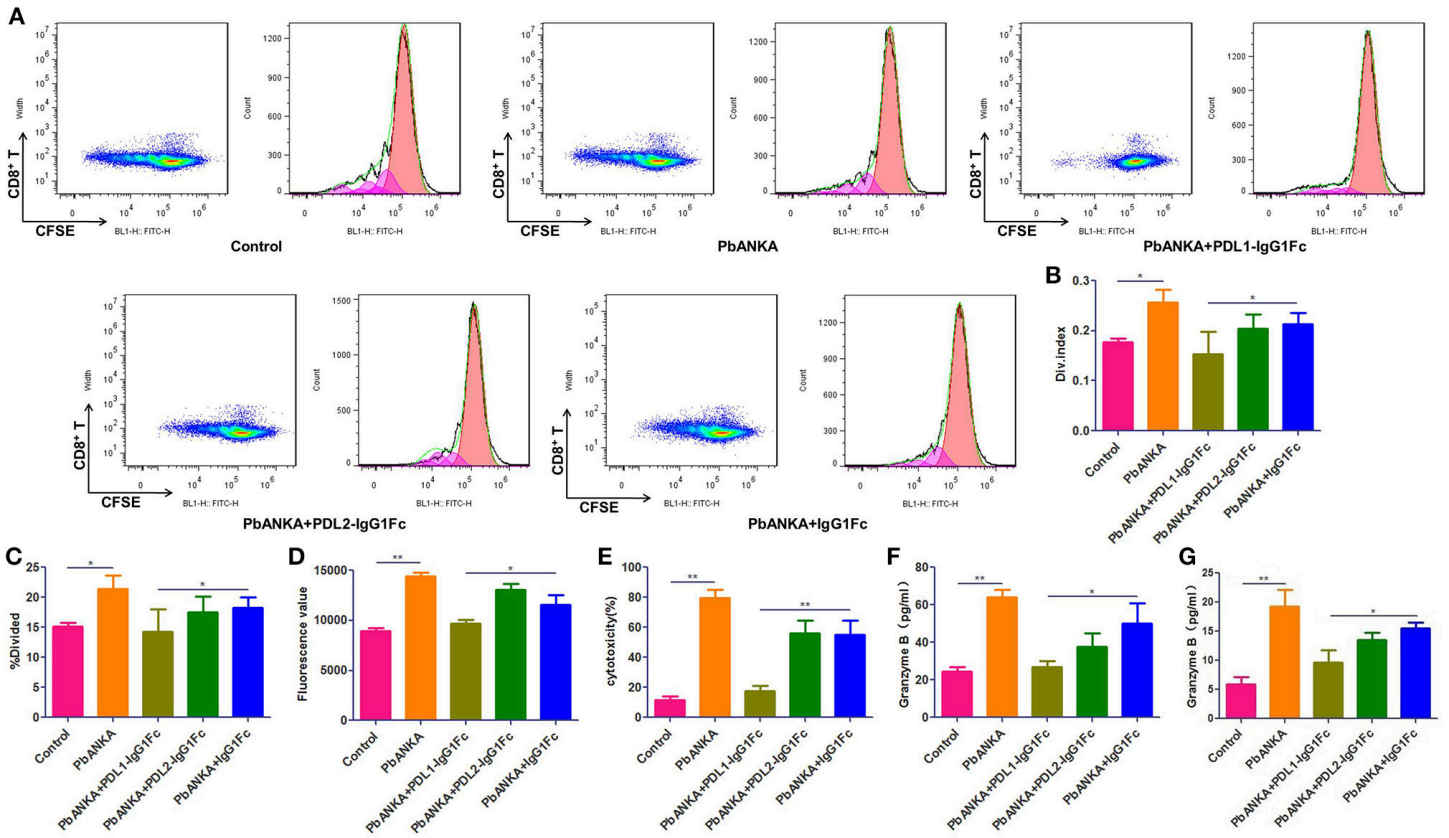
PDL1-IgG1Fc ameliorates BBB disruption by limiting the targeted killing of BMECs by over-reactive CD8^+^ T cells. Splenic CD8^+^ T cells were isolated from different treatment groups, then co-incubation with sub-optimal concentrations of anti-CD3 and anti-CD28 mAbs (0.03 μg/ml) after CFSE staining. After 4 days of culture, CD8^+^ T cells were harvest and assayed for proliferation by flow cytometry. Results of flow cytometry **(A)**, divided index (Div. Index) **(B)** and percentage of divided cell (%Divided) **(C)** analyzed by Flowjo software showed PDL1-IgG1Fc attenuated the proliferation capacity of CD8^+^ T cells. An *in vitro* BBB model was constructed, the amount of FITC-BSA passed through the monolayer of Bend.3 cells into the lower chamber was detected as a sign of permeability of BBB model, the amount of FITC-BSA in lower chamber showed that the cytotoxicity of splenic CD8^+^ T cells to BBB was attenuated in PDL1-IgG1Fc-treated mice **(D)**. Primary BMECs were isolated and co-incubation with pRBCs, IFN-γ and splenic/brain-sequestered CD8^+^ T from different groups of mice. Splenic CD8^+^ T cells cytotoxicity was analyzed by an LDH-release assay **(E)**. Granzyme B secreted by splenic CD8^+^ T cells **(F)** and brain-sequestered CD8^+^ T cells **(G)** was determined by ELISA. Results are from three independent experiments. ^*^ and ^**^ indicate that differences are significant (unpaired *t*-test, 0.01 < *P* < 0.05 and 0.001 < *P* < 0.01, respectively).

As shown in the preceding results, PbA-specific CD8^+^ T cells play a central role in BBB breakdown. Splenic CD8^+^ T cells were isolated and stained with CFSE, then co-incubation with sub-optimal concentrations of anti-CD3 and anti-CD28 mAbs (0.03 μg/ml) for 4 days. The proliferation of T cells was detected by flow cytometry (Figure 5A) and analyzed by Flowjo software, divided index (Figure 5B) and percentage of divided cell (Figure 5C) demonstrated that the proliferation of splenic CD8^+^ T cells was much down-regulated in PDL1-IgG1Fc-treated mice compared with that in control mice (Figures 5A–C). Additionally, to detect the cytotoxicity of activated CD8^+^ T cells to brain endothelial cells, we constructed a BBB model *in vitro* with a Transwell system. Briefly, bEnd.3 cells were seeded into the upper chamber of a 24-well Transwell system, activated with IFN-γ, and then incubated with pRBCs. Activated splenic CD8^+^ T cells from PDLs-IgG1Fc-treated groups were added to the upper chamber. Using FITC-BSA as a permeability tracer, we evaluated the BBB leakage by the diffusion rate of the FITC-BSA from the upper chamber to the lower chamber. By this BBB model, we showed that the cytotoxicity of splenic CD8^+^ T cells to activated ECs was attenuated in the PDL1-IgG1Fc-treated mice compared with that in the PDL2-IgG1Fc-treated and IgG1Fc-treated mice (Figure 5D). To further confirmation the killing effect of activated CD8^+^ T cells on brain endothelial cells, primary BMECs were isolated, then co-incubation with pRBCs, IFN-γ, and splenic/brain-sequestered CD8^+^ T from different groups of mice. LDH assay showed the cytotoxicity of splenic CD8^+^ T cells notable reduction in PDL1-IgG1Fc-treated mice compared with that in control mice (Figure 5E). As CD8^+^ T cell-secreted granzyme B is known to drive CTL-mediated cerebral pathology, our ELISA data showed that the secretion of granzyme B was down-regulated in PDL1-IgG1Fc-treated mice compared with that in control mice, both by splenic (Figure 5F) and brain-sequestered CD8^+^ T cells (Figure 5G). All these results confirmed that PDL1-IgG1Fc protects the BBB against the cytotoxicity of CD8^+^ T cells via limiting T cell proliferation and relieving their targeted killing of BMECs.

### The Effect of PDL1-IgG1Fc on Cytokine Secretion of Macrophage in the Early Stage of PbA Infection

**Figure 6:**
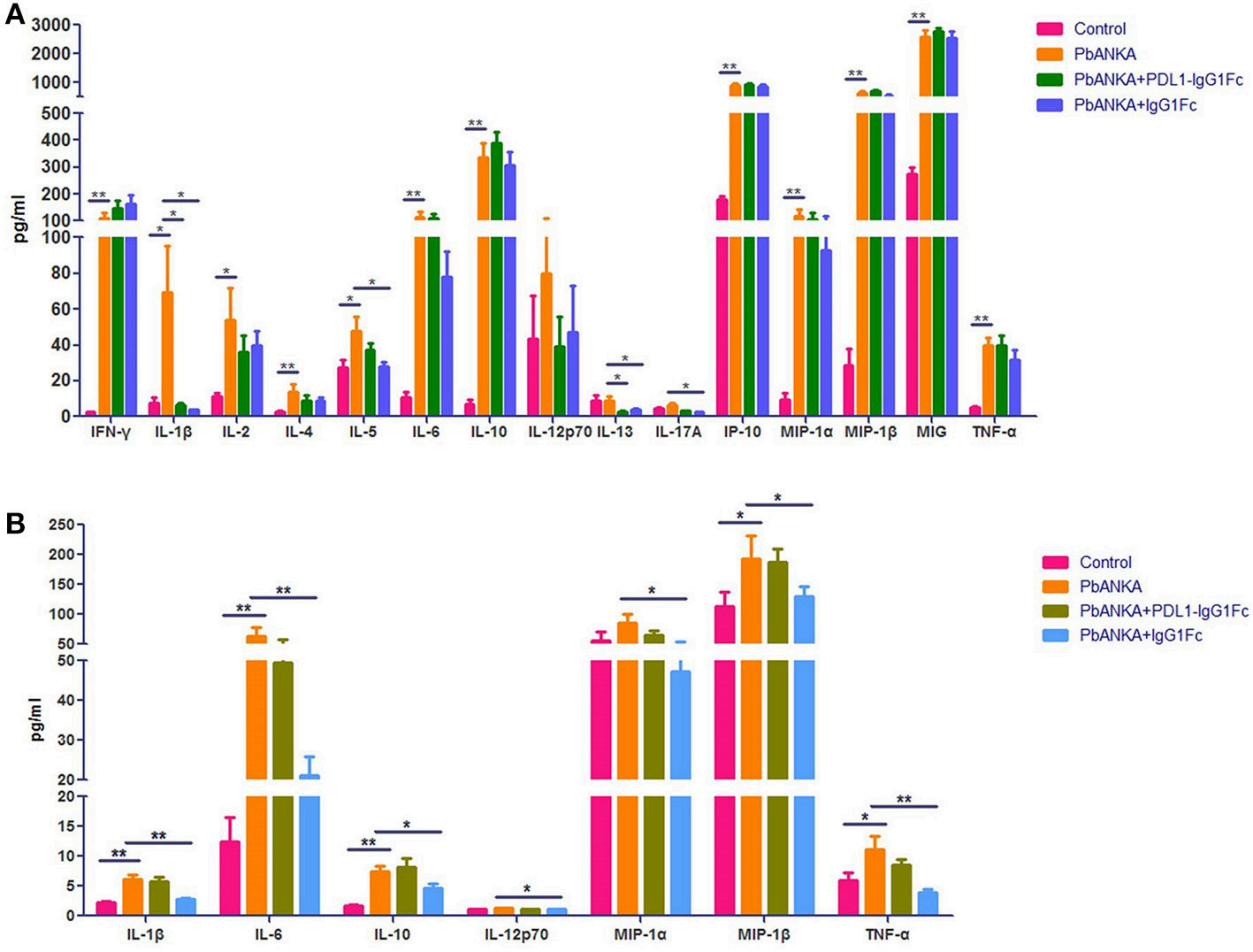
The effect of PDL1-IgG1Fc on cytokine secretion of macrophage in the early stage of PbA infection. C57BL/6 male mice (6 weeks old) were divided into four groups: Control (*n* = 15), PbANKA (*n* = 14), PbANKA + PDL1-IgG1Fc (*n* = 12) and PbANKA + IgG1Fc (*n* = 15). All mice were decapitated at 4 dpi. **(A)** The serum levels of IFN-γ, IL-1β, IL-2, IL-4, IL-5, IL-6, IL-10, IL-12p70, IL-13, IL-17A, IP-10, MIP-1α, MIP-1β, MIG, and TNF-α in these mice were detected by a MILLIPLEX MAP Kit. **(B)** The splenic macrophages of the mice were isolated via a differential adhesion method. The splenic macrophages were respectively planted into 6-well cell culture clusters. After 24 h, the culture supernatants of the macrophages were detected by the MILLIPLEX MAP Kit. Results are from three independent experiments. ^*^ and ^**^ indicate that differences are significant (unpaired *t*-test, 0.01 < *P* < 0.05 and 0.001 < *P* < 0.01, respectively).

We found that PD-1 was also expressed on the surface of macrophages at different polarization statuses (Figure S3). Thus, it was necessary to study the possible influence of PDL1-IgG1Fc on macrophages via the PD-1 pathway. At 4 dpi, the mice from different groups (“Control,” “PbANKA,” “PbANKA + PDL1-IgG1Fc,” and “PbANKA + IgG1Fc”) were euthanized. We detected the inflammatory cytokines secreted by splenic macrophages that were isolated from the mice of different groups, and the serum cytokines of these mice were also detected simultaneously. Among these serum cytokines, the level of IL-1β, one of the most important pro-inflammatory cytokines during PbA infection, was significantly higher in PbANKA group than in control group but was notably reduced by PDL1-IgG1Fc (Figure 6A). Meanwhile, the levels of other serum cytokines exhibited no difference between PbANKA group and PbANKA + PDL1-IgG1Fc group. Interestingly, we found that the IgG1Fc-treated mice showed a significant decline in pro-inflammatory cytokines (including IL-1β, IL-6, IL-12p70, TNF-α, and MIP-1α/β) and in anti-inflammatory cytokine IL-10 compared with PbANKA group, according to the data from *ex vivo* trials (Figure 6B). However, we found that either the pro-inflammatory cytokines (including IL-1β, IL-6, IL-12p70, TNF-α, and MIP-1α/β) or anti-inflammatory cytokine IL-10 in PbANKA + PDL1-IgG1Fc group exhibited no difference compared with PbANKA group. In sum, it appeared that in the early stage of PbA infection, the effect of PDL1-IgG1Fc treatment on cytokine secretion of macrophage was mild and partial.

### PDL1-IgG1Fc Represses Macrophages M1 Polarization and Its Ability to Present Malarial Antigens to CD8^+^ T Cells *in vitro*

**Figure 7:**
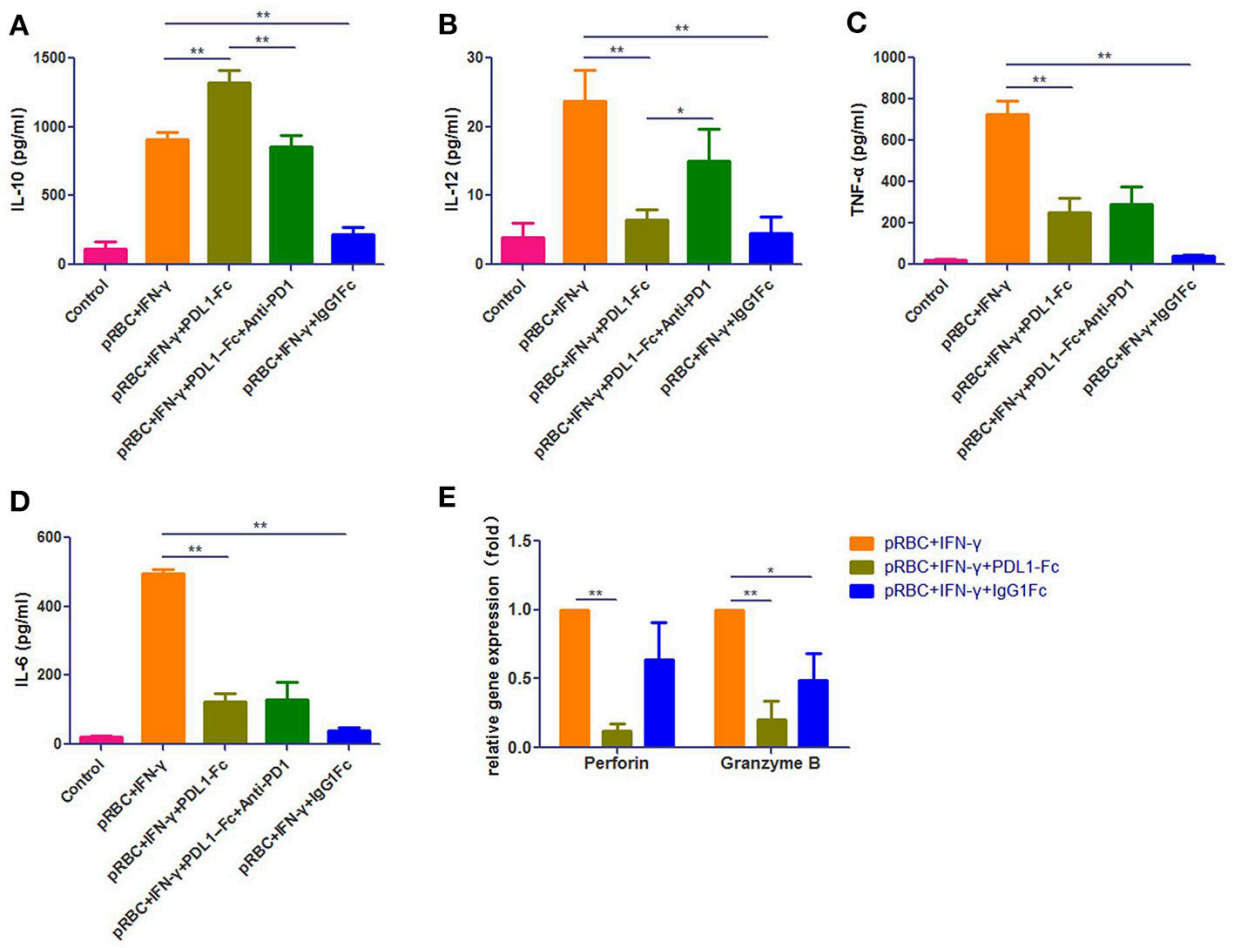
PDL1-IgG1Fc represses macrophages M1 polarization and its ability to present malarial antigens to CD8^+^ T cells *in vitro*. We constructed a macrophage-CD8^+^ T cell co-incubation model to investigate the effects of PDL1-IgG1Fc on macrophages. According to the ELISA results, PDL1-IgG1Fc-treated macrophages showed increased IL-10 secretion **(A)** and decreased secretion in IL-12p70 **(B)**, TNF-α **(C)**, and IL-6 **(D)**, while anti-PD1 mAb was observed to oppose the effect of PDL1-IgG1Fc on IL-10 and IL-12p70. Besides, the perforin and granzyme B mRNA expression levels in CD8^+^ T cells co-incubated with macrophages after being treated with PDL1-IgG1Fc were decreased markedly compared with the levels in the “pRBC+IFN-γ” group **(E)**. These results implied that PDL1-IgG1Fc repressed macrophages M1 polarization and its ability to present malarial antigens to CD8^+^ T cells in vitro. ^*^ and ^**^ indicate that differences are significant (unpaired *t*-test, 0.01 < *P* < 0.05 and 0.001 < *P* < 0.01, respectively).

To further investigate the effects of PDL1-IgG1Fc on macrophages, we constructed a macrophage-CD8^+^ T cell co-incubation model. Immediately before the CD8^+^ T cells were added, we first verified the macrophage polarization influenced by PDL1-IgG1Fc via detecting IL-6, IL-10, IL-12p70, and TNF-α secretion (Figures 7A–D). At this timing, all these cytokines secreted by IgG1Fc-treated macrophages decreased compared with PDL1-IgG1Fc-treated macrophages and “pRBC + IFN-γ” groups; furthermore, PDL1-IgG1Fc-treated macrophages showed increased IL-10 secretion and decreased secretion in IL-6, IL-12p70, and TNF-α, while anti-PD1 mAb was observed to oppose the effect of PDL1-IgG1Fc on IL-10 and IL-12p70. These results implied that PDL1-IgG1Fc repressed macrophages M1 polarization, and the effects of IgG1Fc on macrophages were analyzed in Discussion. Besides, the perforin and granzyme B mRNA expression levels in CD8^+^ T cells co-incubated with macrophages after being treated with PDL1-IgG1Fc were decreased markedly compared with the levels in the “pRBC + IFN-γ” group (Figure 7E), which indicated that the cytotoxicity of the CD8^+^ T cells was suppressed due to the decreased antigen presentation capability of the macrophages.

## Discussion

CM is an acute, lethal neurological complication of *P. f*. infection. Although the precise pathogenesis of CM is still unclear, many studies have demonstrated that the BBB disruption caused by sequestration of pRBCs and vascular immunopathology in brain microvessels is the major reason for the subsequent fatal brain pathology. In this process, parasite-specific brain-infiltrating CD8^+^ T cells mediate BMECs damage through perforin and granzyme B-dependent mechanisms (11, 12, 17). Therefore, it is necessary to maintain a delicate balance between pro- and anti-inflammatory immune responses to control parasitemia properly without inducing immunopathology.

As one of the most efficient immune checkpoint molecules which has been used in the clinical treatment of tumors, PD-1 is a promising immunomodulatory target. In chronic malarial infections, the combined blockade of PDL1 and LAG-3coinhibitory molecules with antibodies accelerates the clearance of non-lethal blood-stage malaria parasites (*P. yoelii*) by improving CD4^+^ T cell functions and increasing protective antibody titers (18); moreover, *Pdcd1*^−/−^ C57BL/6 mice rapidly and completely clear non-lethal malaria parasites (*P. chabaudi*), in contrast to WT mice (19). However, studies on acute lethal malaria parasite (PbA) infection have shown that antibody blockade of PDL1 can lead to T cell hyperactivity, thereby exacerbating ECM, while PDL2 antibody blockade does not affect the immunity against PbA (20, 21). Antibody-mediated blockade of PD-1/PDL1, but not PD-1/PDL2, causes ECM symptoms in ECM-resistant BALB/c mice (20). According to our data, *Pdcd1*^−/−^ C57BL/6 mice exhibit more serious ECM symptoms and a shortened survival time compared with those of WT mice after PbA challenge. All of these results suggest that suppressing the activation and cytotoxicity of CD8^+^ T cells via enhancing the PD-1/PDL signaling pathway to mitigate ECM may be a promising therapy for ECM.

In this study, fusion proteins linking the mouse extracellular domains (ECDs) of PDL1 or PDL2 to the Fc fragment of IgG1 were generated and characterized. Our data showed that the mortality of ECM is significantly decreased in PDL1-IgG1Fc-treated mice compared with PDL2-IgG1Fc or IgG1Fc-treated mice. IL-10 was also decreased in PDL1-IgG1Fc-treated ECM mice compared with the “PbANKA” group, consistent with an epidemiological investigation in Malian children by Lyke et al which showed a significantly elevated level of IL-10 in severe malaria vs. uncomplicated malaria controls (4). Furthermore, the degrees of splenomegaly and body weight reduction were also alleviated in PDL1-IgG1Fc-treated mice compared with the aforementioned two control groups. These results demonstrated that PDL1-IgG1Fc could alleviate the symptoms of ECM. H&E staining showed reducedbrain edema, pRBC sequestration, and leukocyte infiltration in the brains of PDL1-IgG1Fc-treated mice; moreover, the levels of pro-inflammatory cytokines (including IL-1β and IFN-γ) were much lower in these mice than in control mice. More importantly, less BBB disruption was detected in PDL1-IgG1Fc-treated mice by an EB test, implying that PDL1-IgG1Fc can more effectively protect mouse BBB integrity and mitigate subsequent vascular leakage and brain edema compared with control mice. We demonstrated that enhancement of the PD-1 signal pathway by providing a soluble PDL1 ligand could downregulate the excessive pro-inflammatory host immune response, thus reducing the extent of BBB damage and the mortality of ECM.

As is well-known, the targeted killing of BMECs by CD8^+^ CTLs has been proposed to be the major pathogenic process in ECM (22). Our *ex vivo* killing assay also showed that BMECs can serve as non-professional antigen-presenting cells (APCs) and cross-present PbA antigens to activated CD8^+^ T cells originating from ECM mice, which leads to the targeted killing of BMECs by CD8^+^ T cells in a granzyme B-dependent manner, consistent with the reports by Howland et al. (22, 23). More importantly, the pathology caused by CD8^+^ T cells can be markedly repressed by the administration of PDL1-IgG1Fc.

To detect the cytotoxicity change in CD8^+^ T cells, an *in vitro* BBB model using a Transwell system and the bEnd.3 EC line were adopted. Splenic CD8^+^ T cells were isolated from infected mice and incubated with endothelial cells presenting PbA antigens. We found that the killing of ECs by CD8^+^ T cells was attenuated in PDL1-IgG1Fc-treated mice compared with the PDL2-IgG1Fc or IgG1Fc groups. Additionally, the LDH cytotoxicity assay also showed reduced cytotoxicity of activated CD8^+^ T cells to primary BMECs and granzyme B secretion of splenic and brain-infiltrating CD8^+^ T cells in PDL1-IgG1Fc-treated mice. Further studies with CFSE staining suggest that the proliferation of splenic CD8^+^ T cells was inhibited in PDL1-IgG1Fc-treated mice. These results demonstrated that the cytotoxicity of CD8^+^ T cells was reduced in PDL1-IgG1Fc-treated mice due to a stronger inhibitory signal transduction via the PD-1 receptor on the T cells.

Current studies have shown that most PbA-specific CD8^+^ T cells are activated in the spleen (22, 24), and the studies by Hermsen et al. (25) and Amante et al. (26) have shown that ECM and BBB breakdown can be prevented by splenectomy. However, it was also reported that brain endothelial cells were non-specific APC cells that could cross-present parasite antigen to parasite specific CD8^+^ T cells (22, 27). CD8^+^ T cells can be activated *in situ* after accepting malarial antigens cross-presented by BMECs in an MHC-I-dependent manner in brain microvessels (28, 29). Although we still speculate that naïve T cells were activated in the spleen and followed by secondary activation in the brain by antigen-presenting endothelial cells, the contribution ratio of activated CD8^+^ T cells originating from the spleen vs. brain and their subsequent effects may be an interesting direction.

Our studies have shown that PDL1 plays a dominant immunomodulatory role in the ECM model, as PDL1-IgG1Fc, but not PDL2-IgG1Fc, inhibits CD8^+^ T cell proliferation stimulated by anti-CD3 and anti-CD28 mAbs. Moreover, the combination of the two soluble recombinant proteins has no obvious synergistically immunosuppressive effect on CD8^+^ T cells. However, Wykes et al. have shown that PDL2 is essential for protecting against *P. f*. infection, which implies that PDL2, but not PDL1, expression on human blood dendritic cells (DCs) is correlated with reduced parasitemia due to enhanced protective CD4^+^ T cell responses (21). In contrast, our *in vitro* study indicated that PDL1 expression was increased in primary BMECs after being stimulated by IFN-γ and pRBCs, while PDL2 was not changed. In conclusion, these results suggest that these two PD-1 ligands may play different roles in different parasite-host interactions during *plasmodium* infections.

Although CD8^+^ T cell-mediated adaptive immunity is the major contributor to ECM pathology, macrophage-mediated innate immunity also has a key effect on the ECM immunopathological process. After being challenged by PbA, the notable reduction in serum IL-1β in PDL1-IgG1Fc-treated ECM mice suggested that PDL1-IgG1Fc had an anti-inflammatory function in the early stage of the host immune response. However, further detection on cytokines (IL-1β, IL-6, IL-10, IL-12p70, TNF-α, and MIP-1α/β) secreted by splenic macrophages showed no difference between PbANKA + PDL1-IgG1Fc group and PbANKA group. The results may relate to the sampling time; the expression profiles of these cytokines may be diverse in different timings. More importantly, we found that in our *in vitro* experiment, PDL1-IgG1Fc-treated macrophages showed increased IL-10 secretion and decreased secretion in IL-6, IL-12p70, and TNF-α, while anti-PD1 was observed to oppose the effect of PDL1-IgG1Fc on IL-10 and IL-12p70. These data strongly implied that PDL1-IgG1Fc inhibits macrophage M1 polarization, thus limits the pro-inflammatory status in the early stage of ECM. In addition, our further study in the co-incubation model showed that PDL1-IgG1Fc significantly repressed antigen presentation by macrophages to CD8^+^ T cells, as the perforin and granzyme B expression levels in CD8^+^ T cells decreased markedly.

It is indeed unexpected that IgG1Fc has such potent capacity to inhibit cytokine expression of macrophage. A serious of researches reported that the cross-linking between IgG1Fc and Fc receptor on macrophage can influence its polarization, and the effect is dependent on the concentration of IgG1Fc (30–33). In our experiment, the concentration of IgG1Fc is 10 μg/ml, which is set to match the molarity of PDL1-IgG1Fc group. This concentration, according to the researches by Kis-Toth et al. (30) and Gallo et al. (33), is able to drive macrophage to M2-like polarization, which is consistent with our results in general. Indeed, trying to make a thorough explanation of the IgG1Fc effect on macrophage in our work needs more complicated experiments because of multiple influencing factors in the cell culture system and require more time. Importantly, the effect of IgG1Fc on macrophage is not the central work of our manuscript. All in all, the available results have been able to verify our views, and the detailed mechanism researching of IgG1Fc effect on macrophage in our tests may be explored in another story.

In summary, we provide the evidences that a fusion protein containing the murine PDL1-ECD and IgG1Fc fragment can prevent ECM development by appropriately regulating the PD-1/PDL1 signaling pathway. PDL1-IgG1Fc treatment can inhibit antigen presentation between macrophages and CD8^+^ T cells, down-regulate CD8^+^ T cell activation, and its cytotoxicity and thereby reduce BBB disruption and delay the onset of ECM. Our findings may provide a potential direction for the adjunctive immunomodulatory therapy inhuman CM.

## Author Contributions

KY and YZ conceived and designed the experiments. JW, YuL, YS, JL, YL, YH, and XL performed experiments. JW, YuL, and YZ analyzed the data. DJ and SY contributed reagents and materials. JW, YuL, KY, and YZ wrote the paper.

### Conflict of Interest Statement

The authors declare that the research was conducted in the absence of any commercial or financial relationships that could be construed as a potential conflict of interest.
