# Supplementary material for: PDL1 Fusion Protein Protects Against Experimental Cerebral Malaria via Repressing Over-Reactive CD8+ T Cell Responses
Source: Front Immunol. 2019 Jan 14;9:3157. doi: 10.3389/fimmu.2018.03157 (PMC6339951; doi:10.3389/fimmu.2018.03157)
Supplement: Supplementary file 1 [file Presentation_1.pdf]

# *Supplementary Material*

# PDL1 fusion protein protects against experimental cerebral malaria via repressing over-reactive CD8$^+$ T cell responses

**Wang J[1,2,#], Li Y[1,#], Shen Y[1], Liang J[1], Li YH[1], Huang YX[1], Liu XW[1], Jiang DB[2], Yang SY[2], Zhao Y[1,*], Yang K[2,*]**

[1]Department of medical microbiology and parasitology, Fourth Military Medical University, Xi'an, China

[2] Department of immunology, Fourth Military Medical University, Xi'an, China

[#]These authors contributed equally to this work.

***Correspondence:**

Dr. Yang, yangkunkun@fmmu.edu.cn;

Dr. Zhao, zhaoya@fmmu.edu.cn.

## Supplementary Figures

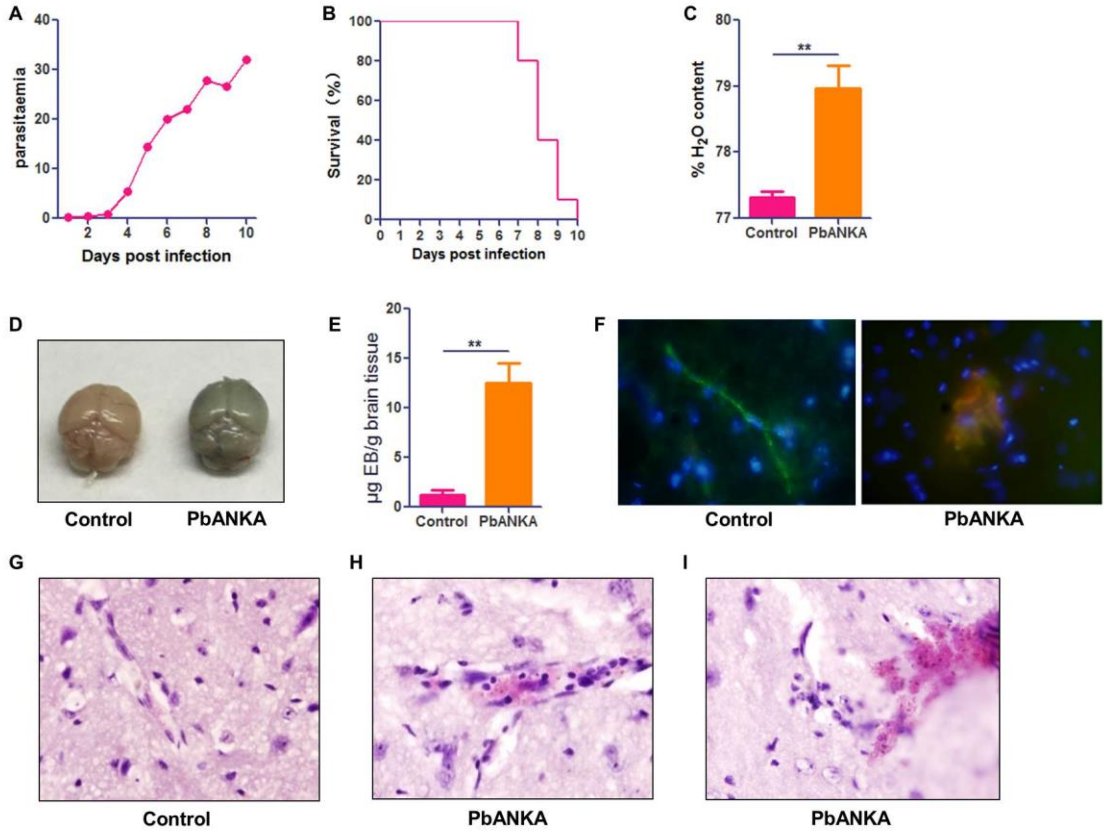


**Supplementary Figure 1. ECM model construction.** C57BL/6 male mice (6-8 weeks old) were infected i. p. with $1\times10^7$ pRBCs. Survival curves (A) and blood parasitemia (B) in WT C57 mice infected with PbA. (C) Quantification of the brain water content from uninfected (left) and PbA-infected (right) mice at 7 dpi revealed evidence of profound vascular breakdown during ECM (n=10). (D) Representative brains from WT (left) and PD-1$^{-/-}$ (right) mice injected i. v. with EB dye at 7 dpi. (E)

Spectrophotometric quantification of EB. (F) Fluorescence of EB revealed vascular breakdown during ECM (red: EB, green: CD31, Blue: nucleus). (G) H&E stained sections from the brains of uninfected (left) and PbA-infected (right) mice 7 dpi demonstrated evidence of increased lymphocyte infiltration (H) and perivascular hemorrhaging (I) in the brain parenchyma during ECM. Data are from three independent experiments. * and ** indicate that differences are significant (unpaired t-test, n =10, 0.01<P<0.05 and 0.001<P<0.01, respectively).

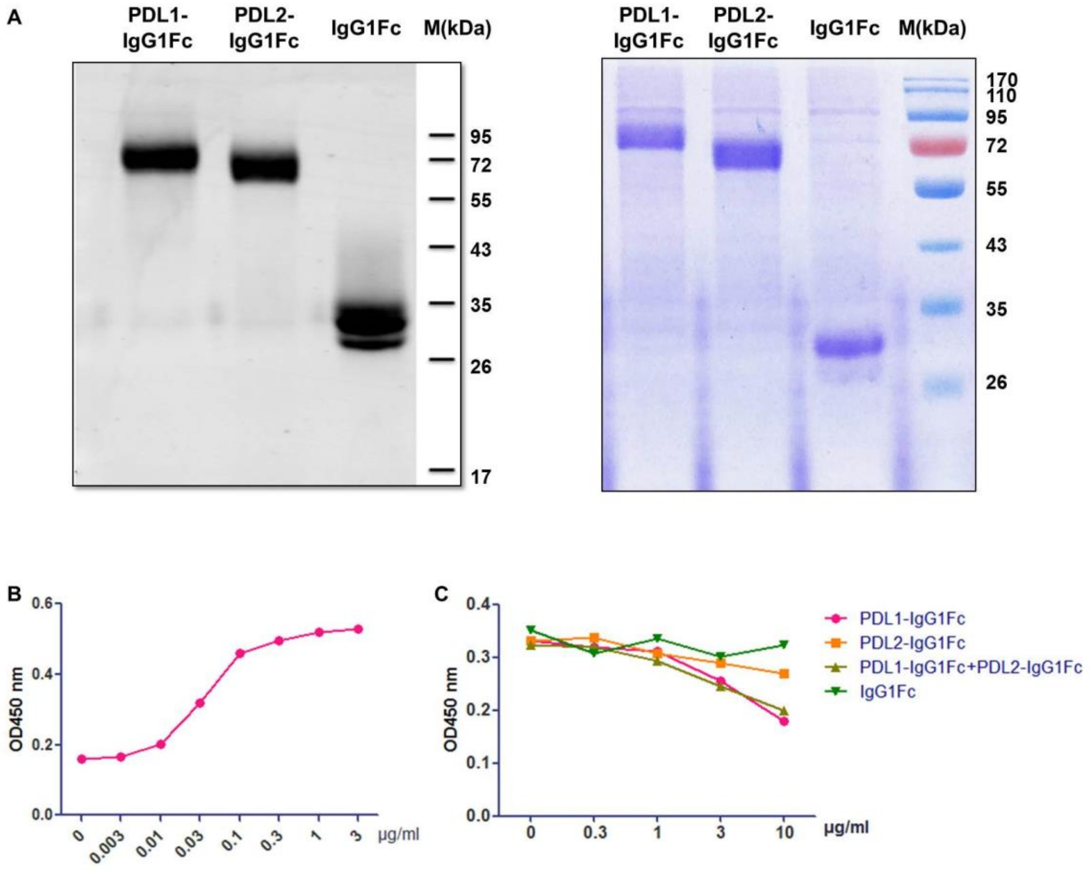


**Supplementary Figure 2. Experimental preparation of PDL1-IgG1Fc and PDL2-IgG1Fc.** (A) Fusion proteins linking the ECD of mouse PDL1/PDL2 to the IgG1Fc were generated and confirmed by WB (left) and SDS-PAGE (right). (B) Splenic $CD8^+$ T cells from WT C57BL/6 mice were stimulated for 96 h with various concentrations of anti-CD3 and anti-CD28 mAbs. (C) Splenic $CD8^+$ T cells from WT C57BL/6 mice were stimulated for 96 h with 0.03 μg/ml of anti-CD3 and anti-CD28 mAbs combined with various concentrations of PDL1/PDL2 fusion protein or IgG1Fc.

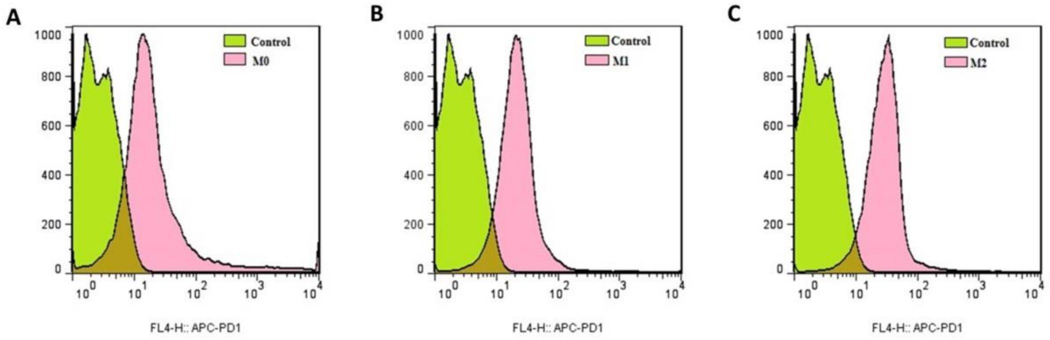


**Supplementary Figure 3. Expression of PD-1 on the surface of macrophages.** To ascertain the PD-1 expression of macrophages with different polarization statuses, we isolated bone marrow-derived macrophages from C57BL/6 male mice (6 weeks old) and polarized them. M1 polarization (B) was induced by IFN-γ (20 ng/ml) and LPS (100 ng/ml). M2 polarization (C) was induced by IL-4 (20 ng/ml). Then, these macrophages were marked with APC-labeled anti-PD-1 and detected by flow cytometry. The results showed that, regardless of its polarization (A-C), PD-1 could be expressed on the surface of macrophages.

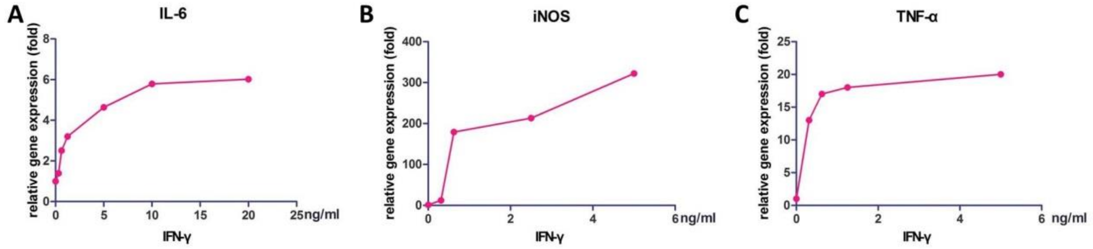


**Supplementary Figure 4. Concentration gradient experiments of IFN-γ to stimulate macrophages.** Aiming to find the proper concentration of IFN-γ that can stimulate macrophages and avoid overreaction, we set eight concentration gradients of IFN-γ (0, 0.3, 0.6, 1.3, 2.5, 5, 10 and 20 ng/ml) to induce macrophages for 24 h. Then, the cytokine (including IL-6 (A), inducible nitric oxide synthase (iNOS) (B), and TNF-α (C)) expression levels of these macrophages were detected by qPCR. Based on the results, we chose the suboptimal concentration of 0.5 ng/ml as the suitable option.
